# Movement kinematic and postural control differences when performing a visuomotor skill in real and virtual environments

**DOI:** 10.1007/s00221-023-06639-0

**Published:** 2023-05-24

**Authors:** K. Brock, S. J. Vine, J. M. Ross, M. Trevarthen, D. J. Harris

**Affiliations:** School of Public Health and Sport Sciences, Faculty of Health and Life Sciences, University of Exeter, St Luke’s Campus, Exeter, EX1 2LU UK

**Keywords:** Virtual reality, Mixed reality, Motor control, Golf

## Abstract

Immersive technologies, like virtual and mixed reality, pose a novel challenge for our sensorimotor systems as they deliver simulated sensory inputs that may not match those of the natural environment. These include reduced fields of view, missing or inaccurate haptic information, and distortions of 3D space; differences that may impact the control of motor actions. For instance, reach-to-grasp movements without end-point haptic feedback are characterised by slower and more exaggerated movements. A general uncertainty about sensory input may also induce a more conscious form of movement control. We tested whether a more complex skill like golf putting was also characterized by more consciously controlled movement. In a repeated-measures design, kinematics of the putter swing and postural control were compared between (i) real-world putting, (ii) VR putting, and (iii) VR putting with haptic feedback from a real ball (i.e., mixed reality). Differences in putter swing were observed both between the real world and VR, and between VR conditions with and without haptic information. Further, clear differences in postural control emerged between real and virtual putting, with both VR conditions characterised by larger postural movements, which were more regular and less complex, suggesting a more conscious form of balance control. Conversely, participants actually reported less conscious awareness of their movements in VR. These findings highlight how fundamental movement differences may exist between virtual and natural environments, which may pose challenges for transfer of learning within applications to motor rehabilitation and sport.

**Supplementary Information:**

The online version contains supplementary material available at 10.1007/s00221-023-06639-0.

## Introduction

There is growing interest in virtual reality (VR) as a tool for training movement skills for applications in areas like rehabilitation and sport (Adamovich et al. 2009; Gray 2017; Neumann et al. 2018; Kim et al. 2019; Michalski et al. 2019; Harris et al. 2020; Wood et al. 2020; Alrashidi et al. 2022). VR offers many new possibilities for controlling the training environment and can be more cost effective than physical training. However, VR replaces the sensory inputs that guide online movement control with computer generated ones. This creates an altered perceptual environment, where inputs are different, uncertain, or missing (Bingham et al. 2001; Harris et al. 2019, 2020; Wijeyaratnam et al. 2019; Giesel et al. 2020). These perceptual deficits include unnatural or conflicting depth cues (e.g., see Wann et al. 1995), limited field of view, missing or inaccurate haptic information, distortions of space, and small rendering lags (see Harris et al. 2019 for review). Crucially, it is unknown how these perceptual deficits impact the control of goal-directed movements. If motor movements are performed differently in VR, it may limit how well skills learned in these environments can transfer back to the real world, which would have significant practical implications. The aim of this study was, therefore, to explore potential movement differences in the execution of a self-paced motor skill (golf putting) between the real world and VR, to understand how virtual environments may influence the control of action.

While VR technologies continue to develop at a rapid pace, the lack of realism, or even total absence, of haptic information may be one of the largest hurdles for motor learning applications. Previous work has examined how the absence of end-point haptic feedback may impair simple reaching movements, which tend to become more simplified or exaggerated (Goodale et al. 1994; Whitwell et al. 2015). Broadly similar, although somewhat inconsistent, results for reach-to-grasp movements in VR have subsequently been found (Viau et al. 2004; Gerig et al. 2018; Furmanek et al. 2019). For instance, Magdalon et al. (2011) found that when reaching to grasp objects in a virtual environment, reaching and grasping coordination was preserved but movements were slower and had longer deceleration times compared to the physical environment. This is despite receiving some end-point feedback in VR via a haptic glove. A review by Harris et al. (2019) suggested that the combination of unnatural cues to depth and impaired or absent haptic information could bias users towards a more deliberate, conscious mode of action control, that makes greater use of the ventral visual stream (due the added uncertainty of visual cues and lack of haptics), as opposed to normal online control of visually guided action by the dorsal stream (Goodale and Milner 1992; Goodale 2017). The slower more exaggerated movements observed in reach-to-grasp studies are consistent with this hypothesis, but relatively few studies have examined more complex whole-body movements like sporting skills.

Existing studies that have compared more complex movements between real and VR environments have indicated that differences may be present here too. For instance, Bufton et al. (2014) compared children’s table tennis shots in the real world with three gaming environments (Nintendo Wii, Xbox Kinect, Sony Move), finding that the wrist angle range, elbow angle range, hand path distance, average speed, and maximum speed of shots were all higher than in the real world. These results are again consistent with more exaggerated, consciously controlled movements in virtual environments. Differences in the success of balance, grasping, and throwing skills in real and VR settings have also been reported by Pastel et al. (2020), particularly when body visualization was absent from the VR environment.

A study by Naylor et al. (2021) explicitly examined the role of haptic feedback in mixed reality golf putting. The authors compared conditions where contact between a virtual putter with a virtual ball either was or was not paired with contact between a physical putter and a real ball. The authors predicted that integrating sensory information from different modalities could resolve perceptual ambiguities and improve action execution (Lalanne and Lorenceau 2004), but the haptic condition was actually detrimental to performance in VR, resulting in larger radial errors of the (virtual) ball from the hole. The authors suggested that minor incongruences between the visual and tactile cues could be responsible for this effect.

Consequently, we still have a limited understanding of how movement skills are affected by VR, how the presence or absence of haptic feedback impacts movement, and whether movements tend to be more exaggerated and consciously controlled in the virtual environment. We aimed to address these questions in the current work. As discussed, several previous studies have examined simple reaching tasks in VR, but so far there has been relatively little investigation of more complex movements that are representative of real-world applications of VR training (e.g., sport and rehabilitation). In addition, studies to date have focused on snapshot or summary measures of movement, like peak reach velocity or mean joint angles, rather than more continuous movement profiles. Therefore, as well as studying a more complex movement skill in VR, we aimed to supplement some traditional movement measures with more continuous comparisons of movements made possible by statistical parametric mapping (Friston et al. 2011).

Whether or not VR induces movements that are different from the real world will depend, in part, on the specifics of the skill and the technologies used. Consequently, making generalisations across tasks about the effect of VR is difficult. Therefore, our intention here was to begin to examine whether there are particular *patterns* of movement that suggest fundamentally different control strategies, such as more conscious and exaggerated movement, as has been suggested by Harris et al. (2019). We, therefore, assessed two aspects of movement during the golf putting task, the swing of the putter and the control of posture, and whether they differed between the real world and VR, both with and without haptic feedback.

We compared the following variables between VR, VR Haptic, and real-world conditions:

(i) Summary measures of acceleration and jerk of the putter swing, previously used to characterise expertise (Mackenzie and Evans 2010; Sim and Kim 2010; Moore et al. 2012) and variance in the sagittal plane (i.e., perpendicular to the downswing);
(ii) Continuous time-series of putter swing accelerations using statistical parametric mapping (Friston et al. 2011);
(iii) The amplitude of postural sway and measures of total postural adjustments (centre of pressure path length and area of the 95% confidence ellipse of centre of pressure);
(iv) The complexity (entropy) of postural sway to index more automatic (i.e., less consciously processed) postural control (Borg and Laxåback 2010);
(v) Self-reported conscious awareness of movement.

In line with findings from the previously discussed studies on reaching in VR, and the hypotheses outlined in Harris et al. (2019), it was predicted that club swings in VR would tend to be slower and more controlled, postural adjustments would be larger and more consciously controlled, and participants would be more consciously aware of their movements.

## Methods

### Design

We used a repeated measures design in which participants completed three putting conditions in a counterbalanced order determined by a Latin squares design. The three conditions were: (1) real-world putting; (2) VR putting; and (3) VR putting with haptic feedback (VR Haptic). The primary outcome measures were clubhead acceleration and centre of pressure data and the secondary outcome measure was self-reported conscious movement processing.

### Participants

Twenty one undergraduate student participants were recruited to take part in the study (20 M, 1F; *M*_age_ = 21.22 ± 1.06). All participants were novice golfers, defined as individuals with no official golf handicaps or prior formal golf putting experience (as in Moore et al. 2012; Harris et al. 2020). One participant had no data for accelerometery variables and five had no data for the pressure plate, due to recording errors. A series of power curves (see: https://osf.io/h8az7/) for linear mixed effects models were generated using the simr package for R (Green and MacLeod 2016). These analyses indicated that given 20 trials per participant we had > 90% power to detect small differences in accelerations down to 1 m/s^2^ and ~ 80% power for differences as low as 0.5 m/s^2^. Therefore, even with some data loss for some variables we were still well powered to detect even small between-condition differences. Ethical approval was provided by the University Ethics Committee before data collection and participants gave written informed consent prior to taking part.

### Tasks

#### VR golf putting

**Fig. 1:**
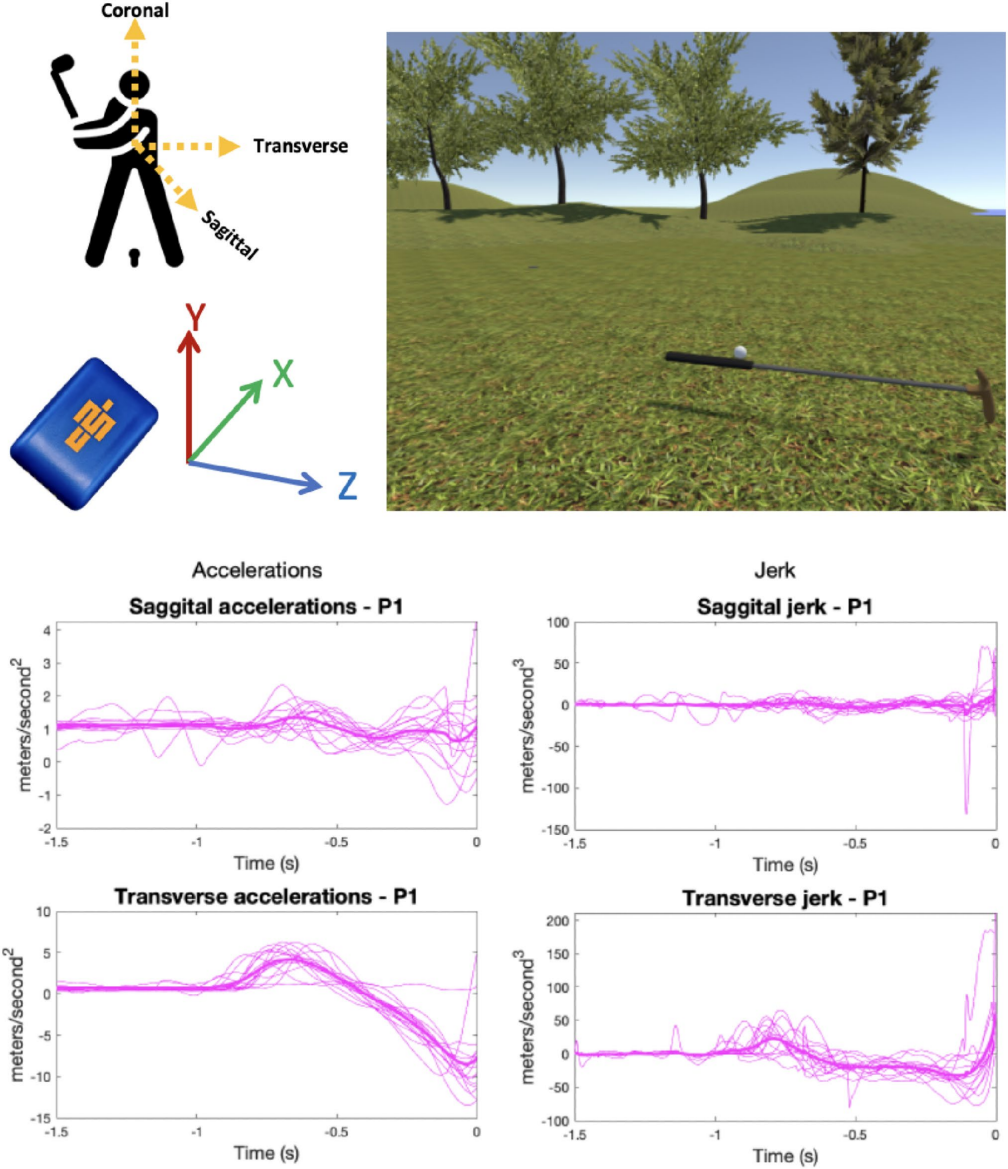
The VR golf environment (top right) and examples of individual trial signals for clubhead acceleration and jerk in a single participant (bottom)

The VR golf putting simulation (as used in Harris et al. 2020; see Fig. 1) was developed using the gaming engine Unity 2019.2.12 (Unity technologies, CA) and C#. The simulation was displayed using an HTC-Vive Pro Eye (HTC, Taiwan), a 6-degrees of freedom, consumer-grade VR-system which allows a 360° environment and 110^o^ field of view. Graphics were generated with an HP EliteDesk PC running Windows 10, with an Intel i7 processor and Titan V graphics card (NVIDIA Corp., Santa Clara, CA). The VR putter was animated by attaching a Vive tracker to the head of a real golf club. Participants putted from 10ft (3.05 m) to a target the same size and shape (diameter 10.80 cm) as a standard hole and were instructed to land the ball as close as possible to the target (the ball did not drop into the hole). Auditory feedback mimicking the sound of a club striking a ball was played concurrently with the visual contact of the club with the ball. The environment also featured ambient environmental noise to simulate a real-world golf course. Testing of the construct validity of an earlier version of this task for simulating putting is described in Harris et al. (2021).

#### Real golf putting

Real-world putts were taken on an indoor artificial putting green from a distance of 10 ft (3.05 m) to a target of diameter 10.80 cm (regulation hole size). Participants were not given verbal feedback about the radial errors of puts, but the landing position of the ball was apparent (as it was in VR). Participants used a Cleveland Classic Collection HB 1 putter, and standard size (4.27 cm diameter) white golf balls.

### Measures

#### Clubhead accelerometery variables

Accelerations of the clubhead of the putter were recoded using a lightweight (9.5 g) inertial measurement unit (Blue Trident; Vicon, Auckland, New Zealand) using low-*g* (± 16 g) triaxial accelerometers, capable of recording up to 1125 Hz. The accelerometer was attached to a consistent location on the back of the putter. Acceleration data were processed using bespoke analysis scripts in MATLAB (R2019a; Mathworks, US) (available from: https://osf.io/h8az7/). Imported accelerometery files were first resampled to ensure a consistent recording rate then filtered with a 2nd order, 3 Hz Butterworth low pass filter to reduce noise (Mayagoitia et al. 2002). Each putting motion was identified from spikes in the y-axis of the accelerometer signal (i.e., the vertical direction corresponding to coronal plane of the body). The pre-contact phase of the swing was extracted for each putt where this spike could be reliably identified and metrics for the forward (i.e., along the transverse plane of the body) and sideways (i.e., sagittal plane of the body) movement of the putter was calculated.

We then calculated mean values for transverse and sagittal accelerations at moment of impact, variance in sagittal accelerations during the swing, and mean transverse and sagittal jerk (Jerk = ΔAcc/ΔTime). Continuous data across the whole of the pre-contact swing for transverse and sagittal accelerations and jerk were also extracted for statistical parametric mapping analysis. These measurements were chosen based on previous work identifying the relationship between putting expertise and clubhead accelerations (Mackenzie and Evans 2010; Sim and Kim 2010; Moore et al. 2012). Reduced transverse and sagittal accelerations are generally indicative of better putter control so these variables, as well as their derivative jerk, were recorded.

#### Pressure plate variables

Centre-of-pressure (COP) data, which reflect the weighted average of pressure applied by the feet onto the support surface (sampled at 300 Hz), were recorded using a Materialise Footscan 9 pressure plate (488 × 325 mm active sensor area; Materialise Ltd, Rotheram, UK). The location of the COP on the pressure plate, expressed in 2-dimensional coordinates (x,y), was recorded and low-pass (5 Hz) filtered offline with a second-order Butterworth filter (as in Ellmers et al. 2021; Ellmers et al. 2022). Postural control variables (sway amplitude, complexity of sway, COP path length, and COP ellipse area) were then calculated for transverse (y) and sagittal (x) directions. Putting trials on the pressure plate were manually segmented based on foot lifts made by the participant prior to each putt.

##### Postural sway amplitude

To assess the magnitude of changes in balance during putting we calculated the amplitude of COP adjustments, using the root-mean-square (RMS) of COP (with respect to the COP mean position; Zaback et al. 2019; Ellmers et al. 2022).

##### Complexity of postural sway

Complexity of postural sway was assessed by calculating sample entropy (SampEn) of COP data. For static tasks, higher SampEn values reflect greater complexity and irregularity of postural adjustments characteristic of more automatic postural control (Borg and Laxåback 2010). High-complexity of postural sway generally manifests as more frequent smaller adjustments to posture that happen automatically to maintain balance, whereas lower complexity is typical of larger but less frequent (over)adjustments, such as when we are fatigued or consciously controlling movement (Roerdink et al. 2011; Jie et al. 2023). As per previous research (Lake et al. 2002; Roerdink et al. 2011) pressure plate data were down-sampled to 100 Hz when calculating SampEn.

##### COP path length

To measure total postural movement during the putt we calculated COP path length, which represents the distance travelled in the 2-dimensional plane of the pressure plate. Path length was calculated from the cumulative sum of the filtered COP signal (in mm; Ellmers et al. 2020), and therefore indexes when the centre of balance moved around the pressure plate to a greater degree.

##### Ellipse area

In addition to the total path length, we also quantified the area covered by the COP as a 95% confidence interval (CI) ellipse around the COP path, calculated from a principal components analysis of the COP position data (as described in Duarte 2015).

#### Conscious movement processing (CMP) questionnaire

Conscious movement processing was measured using a version of the conscious motor processing subscale of the Movement Specific Reinvestment Scale (MSRS; Orrell et al. 2009) specifically adapted for use in a golf putting task (seeCooke et al. 2011; Vine et al. 2013). Participants were asked to reflect on the previous block of trials in relation to 6 items, for example “*I reflected on my technique”* and *“I was aware of the way my body was working”*. Items were scored on a 5-point Likert scale (1 = Strongly Disagree; 5 = Strongly Agree). Participants completed the scale after each condition (Real-world putting; VR putting; and VR putting with haptic feedback) and a mean score for the six items was included in subsequent analysis.

### Procedure

Participants attended the lab on a single occasion for approximately 1 h. All participants provided written informed consent before the start of the study. Participants were given one familiarisation putt when in the real world, VR with a real-world ball, and VR with no real-world ball. They then completed 20 putts in each condition, in an order that was counterbalanced across participants. Participants were asked to aim to land the ball as near to the ‘hole’ as possible. After each condition, participants completed the conscious movement processing questionnaire.

### Data analysis

#### Statistical parametric mapping

Statistical parametric mapping (SPM) is a form of inferential testing for continuous time series data sets that controls the type 1 error rate by treating data as a topographical map and then correcting for clusters of values that cross a significance threshold (Friston et al. 2011). SPM was originally devised for brain imaging data (Friston et al. 2011) but has since been applied to other biological time series like EMG and joint biomechanics (Pataky 2010; Robinson et al. 2015). These types of data pose an analysis challenge when we wish to compare entire time series recordings between groups without inflating the type 2 error rate with overly conservative corrections for a large number of pairwise comparisons across timeseries. As biological data tend to exhibit spatiotemporal correlation (local smoothness) differences occur not at a single timepoint but in clusters. By treating the differences between timeseries as a topographical map (in two, three, or more dimensions) the error rate can be controlled. Conceptually the analysis is similar to a *t*-test, but an output statistic is calculated for each timepoint (creating the statistical ‘map’) and then adjusted based on local covariance. SPM enables the full data field to be examined in a non-directional hypothesis test without any ad-hoc assumptions regarding the spatiotemporal foci of interest. SPM also enables movement data to remain in the biomechanically meaningful sampling space (Pataky 2010). See Pataky (2010) or Robinson, Vanrenterghem and Pataky, (2015) for further explanation. The open source spm1D (v4.0) Matlab package (https://spm1d.org/index.html) was used to calculate the scalar test statistic SPM{F} to compare swing kinematics between the conditions. The resultant p-value indicates the probability that smooth, random continua would produce a supra-threshold cluster in the map as broad as the observed cluster.

#### Statistical analysis

Data analysis was performed in RStudio v1.0.143 (R Core Team 2017). The data were first screened for outlying values more than 3 standard deviations from the mean (Tabachnick & Fidell 1996), which were replaced with a Winsorized score by changing the outlying value to a value 1% larger (or smaller) than the next most extreme score. A series of linear mixed effects models (LMMs; fitted using restricted maximum likelihood in the lme4 package (Bates et al. 2014)) were used to examine the effect of putting condition on putter swing variables. Model fit checks were performed using the ‘performance’ package (Lüdecke et al*.* 2021) and can be accessed from the supplementary materials (https://osf.io/h8az7/). When reporting results of the LMMs, we followed the effect size rules of thumb outlined in Acock (2014) that standardised beta effect sizes can be interpreted similarly to *r* (i.e., < 0.2 is weak, 0.2–0.5 is moderate, and > 0.5 is strong). In essence, a standardised beta of 0.5 indicates that a one standard deviation change in the predictor variable equates to a half standard deviation change in the outcome variable.

## Results^1^

### Downswing acceleration at impact

#### Transverse

**Fig. 2:**
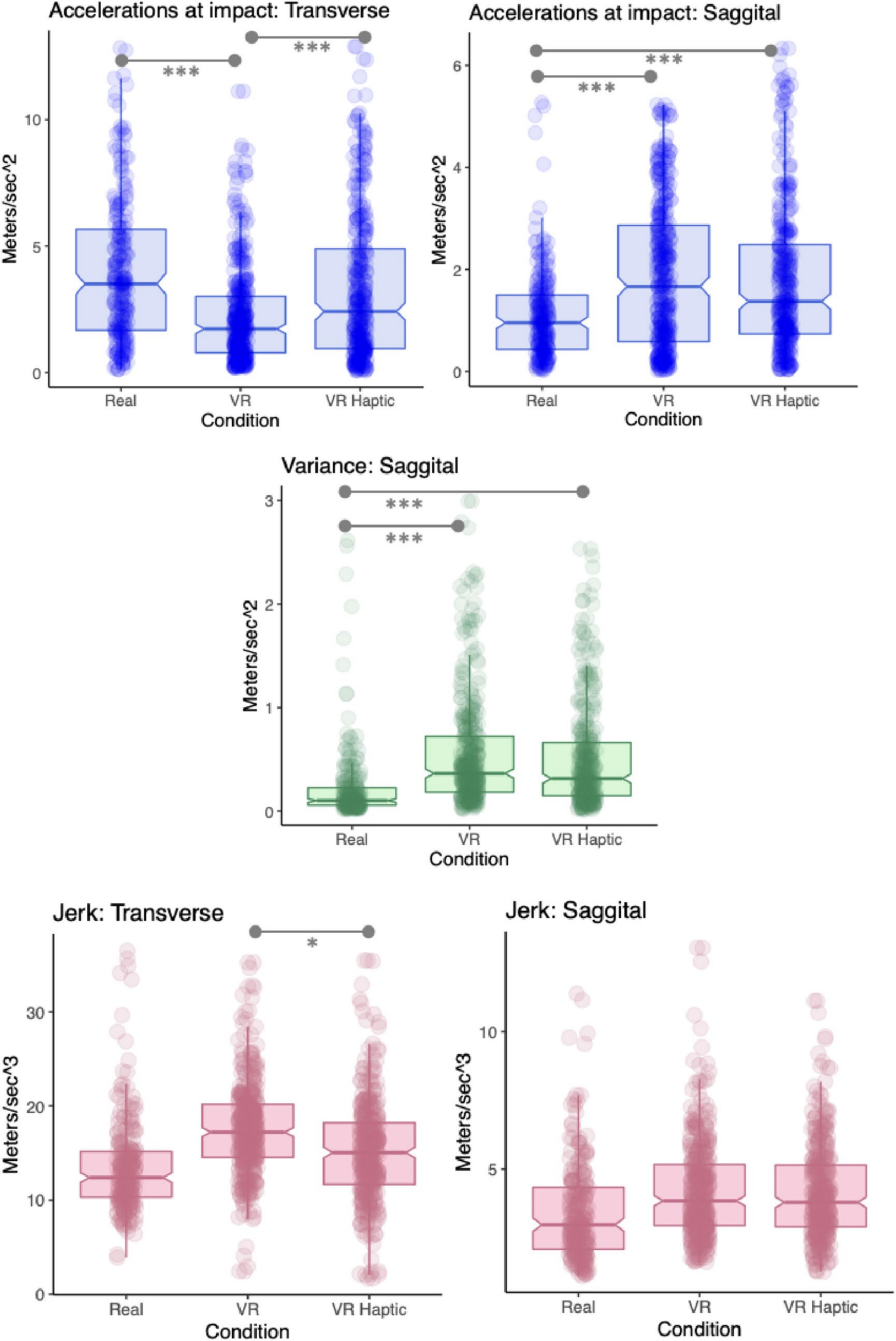
Boxplots with overlaid data points for acceleration and jerk data. *p < .05; **p < .01;***p < .001

We fitted a linear mixed effects model to explain clubhead accelerations in the transverse (forward) plane at impact (Fig. 2, top and Table 1). The best fitting model included random intercepts for the participant factor. Within this model, the effect of VR (*p* < 0.001; std. beta =  – 0.55) and VR Haptic (*p* = 0.05; std. beta =  – 0.16) were both significant. Bonferroni corrected pairwise comparisons indicated higher accelerations in the real world compared to VR (*p* < 0.001) and in VR Haptic compared to VR (*p* < 0.001), but no difference real world and VR Haptic (*p* = 0.15).

**Table 1:** Summary of model coefficients and variance explained

Dependent variable	Total *R*^2^	Marginal *R*^2^	Intercept (Condition = real world)	Group effects	
Clubhead kinematic variables					
Transverse accelerations at impact	0.21	0.05	3.69 (95% CI [3.12, 4.27], *p* < .001)	VR	*β* = – 1.47, 95% CI [ – 1.87, – 1.06]
				VR Haptic	*β* = – 0.42, 95% CI [ – 0.83, – 1.30e-04]
Sagittal accelerations at impact	0.43	0.07	0.95 (95% CI [0.55, 1.35], *p* < .001)	VR	*β* = 0.90, 95% CI [0.71, 1.08]
				VR Haptic	*β* = 0.78, 95% CI [0.60, 0.97]
Sagittal variance	0.38	0.17	2.21 (95% CI [ – 2.47, – 1.94], *p* < 0.001)	VR	*β* = 1.20, 95% CI [1.04, 1.36]
				VR Haptic	*β* = 0.95, 95% CI [0.79, 1.11]
Transverse jerk	0.48	0.06	14.58 (95% CI [12.42, 16.75], *p* < .001)	VR	*β* = 3.36, 95% CI [0.67, 6.05]
				VR Haptic	*β* = 0.85, 95% CI [ – 2.28, 3.98]
Sagittal jerk	0.36	0.01	3.82 (95% CI [3.08, 4.56], *p* < .001)	VR	*β* = 0.53, 95% CI [ – 0.40, 1.45]
				VR Haptic	*β* = 0.33, 95% CI [ – 0.64, 1.29]
Postural control variables					
Transverse postural sway	0.46	0.04	9.49 (95% CI [7.49, 11.49], *p* < .001)	VR	*β* = 2.63, 95% CI [0.57, 4.69]
				VR Haptic	*β* = 2.87, 95% CI [ – 4.70e-03, 5.74]
Sagittal postural sway	0.50	0.04	11.71 (95% CI [9.31, 14.11], *p* < .001)	VR	*β* = 4.73, 95% CI [0.74, 8.73]
				VR Haptic	*β* = 3.00, 95% CI [ – 0.57, 6.58]
Transverse postural sway complexity (SampEn)	0.93	0.09	7.39 (95% CI [5.56, 9.21], *p* < .001)	VR	*β* = 1.75, 95% CI [0.74, 2.76]
				VR Haptic	*β* = 1.73, 95% CI [0.68, 2.77]
Sagittal postural sway complexity (SampEn)	0.96	0.10	8.92 (95% CI [6.51, 11.34], *p* < .001)	VR	β = 2.77, 95% CI [1.46, 4.09]
				VR Haptic	β = 2.04, 95% CI [0.73, 3.35]
COP path length	0.61	0.16	139.90 (95% CI [119.62, 160.18], *p* < .001)	VR	β = 106.62, 95% CI [65.28, 147.96]
				VR Haptic	β = 85.59, 95% CI [30.70, 140.47]
COP ellipse area	0.60	0.05	7.12 (95% CI [6.77, 7.47], *p* < .001)	VR	β = 0.59, 95% CI [0.32, 0.85]
				VR Haptic	β = 0.35, 95% CI [ – 0.04, 0.74]
Self-report					
Conscious movement processing	0.76	0.12	20.28 (95% CI [18.98, 21.58], *p* < .001)	VR	β = − 2.34, 95% CI [− 3.30, − 1.38]
				VR Haptic	β = − 2.06, 95% CI [ – 3.02, – 1.10]

#### Sagittal

A LMM predicting sagittal (lateral) acceleration at contact (with participant as random effect) indicated that the effect of both VR (*p* < 0.001; std. beta = 0.65) and VR Haptic (*p* < 0.001; std. beta = 0.57) was statistically significant and large. Bonferroni corrected comparisons confirmed higher accelerations in both VR (*p* < 0.001) and VR Haptic (*p* < 0.001) compared to the real world, but not between VR and VR Haptic (*p* = 0.50).

### Sagittal variance

We fitted a LMM to predict the log of variance in lateral accelerations during the swing with random intercepts for participants (see Fig. 2, middle and Table 1). Within this model, the effect of both VR (*p* < 0.001; std. beta = 0.39) and VR Haptic were statistically significant (*p* < 0.001; std. beta = 0.31). Bonferroni corrected pairwise comparisons indicated significantly higher variances in both VR (*p* < 0.001) and VR Haptic (*p* < 0.001) compared to the real world. VR also had higher variance than VR Haptic (*p* = 0.001).

### Jerk

#### Transverse

A LMM with random slopes and intercepts for participants showed that the effect of VR on jerk in the transverse plane was statistically significant and large (*p* = 0.01; std. beta = 0.61) but the effect of VR Haptic was non-significant (*p* = 0.60; std. beta = 0.15). Bonferroni-corrected pairwise comparisons indicated a significant difference in transverse jerk between VR and VR Haptic (*p* = 0.01), but not between real world and VR (*p* = 0.07), or between VR Haptic and real world (*p* = 1.00).

#### Sagittal

A LMM with random slopes and intercepts for participants indicated that the effect of both VR Haptic (*p* = 0.51; std. beta = 0.18) and VR (*p* = 0.27; std. beta = 0.29) on sagittal jerk were statistically non-significant.

### Statistical parametric mapping analysis

#### Transverse

**Fig. 3:**
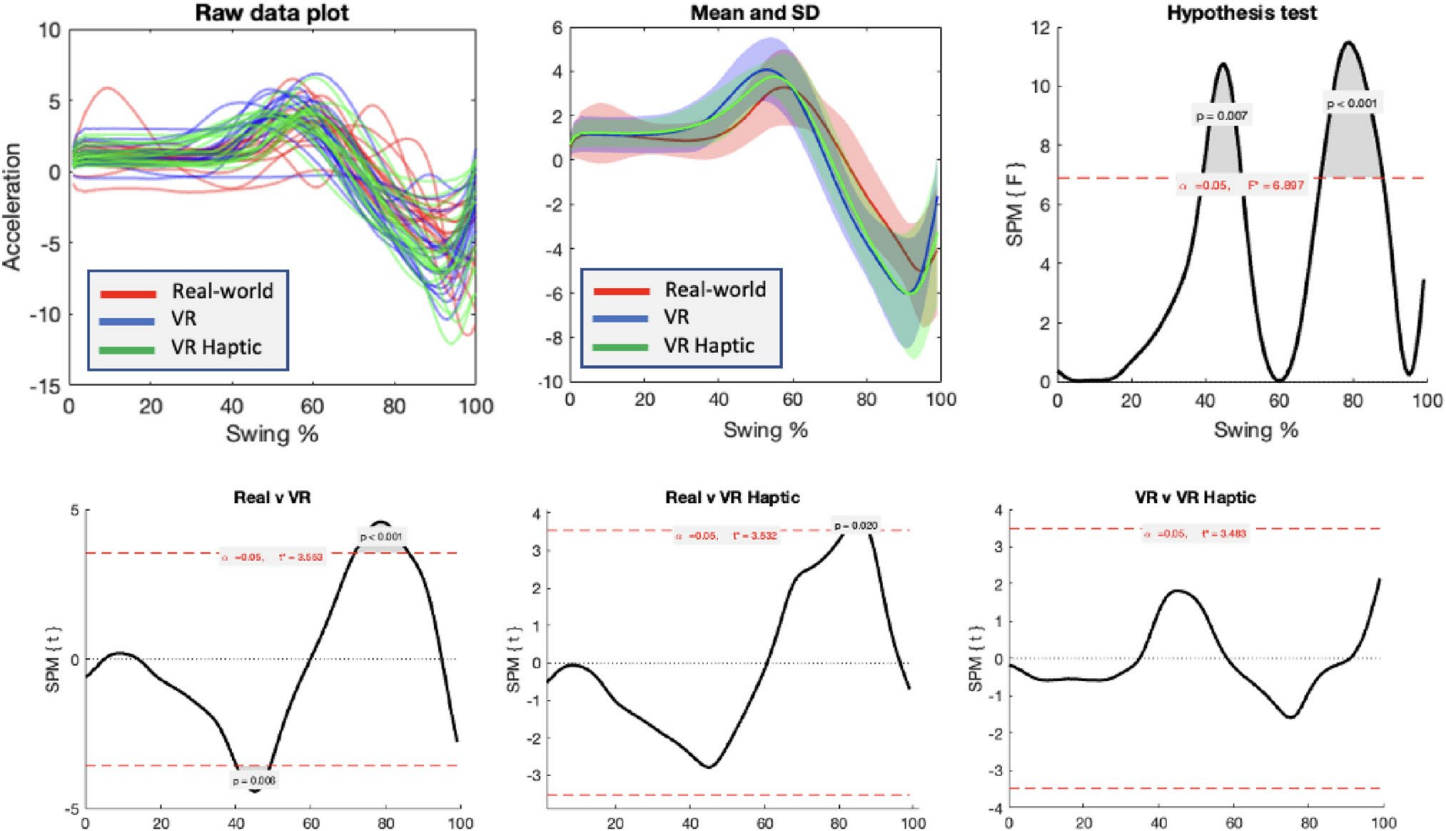
Results of the SPM{F} and SPM{t} analyses for forward swing of the putter (transverse plane). The first two panels show the raw and mean acceleration signals, and panels four to six show the clusters of the topographical map that crossed the corrected alpha threshold

For the transverse plane of the swing, SPM{F} analysis indicated differences at two topographical locations. These clusters (see Fig. 3, top right) corresponded to accelerations around the completion of the backswing of the club (*p* = 0.007) and the rate of the downswing of the club (*p* < 0.001). Pairwise SPM{t} indicated that there were clear differences at these two locations for real world compared to VR (*p* = 0.008 and *p* < 0.001), and a narrowly significant difference at the downswing for real world versus VR Haptic (*p* = 0.02), but no differences between VR and VR Haptic (see Fig. 3, bottom panels). The nature of these differences displayed in Fig. 3 (top middle panel) suggests that the backswing of the club was more exaggerated for both VR conditions (greater and earlier accelerations at ~ 50% of the swing) and the downswing was more pronounced (greater and earlier accelerations at ~ 80% of the swing).

#### Sagittal

**Fig. 4:**
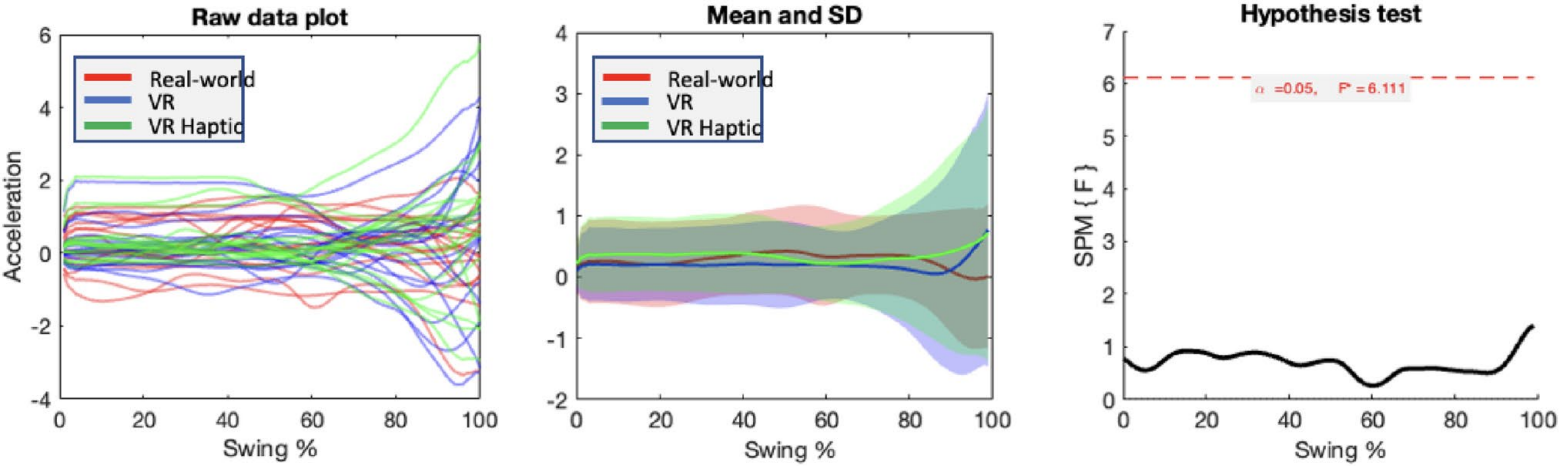
Results of the SPM{F} analyses for sideways swing of the putter (sagittal plane), showing raw (left) and mean (middle) acceleration signals, and the test values of the statistical parametric map (right)

For the sagittal plane (see Fig. 4), there were no clusters that crossed the significance threshold, suggesting no points in the swing were different between conditions.

### Postural sway amplitude

#### Transverse

A LMM to predict postural sway in the transverse plane with condition and participant as random effects showed that the effect of VR was statistically significant and moderate (*p* = 0.01; std. beta = 0.42) but the effect of VR Haptic was statistically non-significant (*p* = 0.05; std. beta = 0.46). Following Bonferroni-corrections for pairwise comparisons, there were no differences between any pairs of conditions (*p*s > 0.11).

#### Sagittal

A LMM to predict sagittal sway amplitude with condition and participant as random effects showed that the effect of VR was narrowly significant (*p* = 0.02; std. beta = 0.49), but VR Haptic was not (*p* = 0.10; std. beta = 0.31). Bonferroni-corrected pairwise comparisons indicated no differences between any pairs of conditions (*p*s > 0.12).

### Postural sway complexity

#### Transverse

**Fig. 5:**
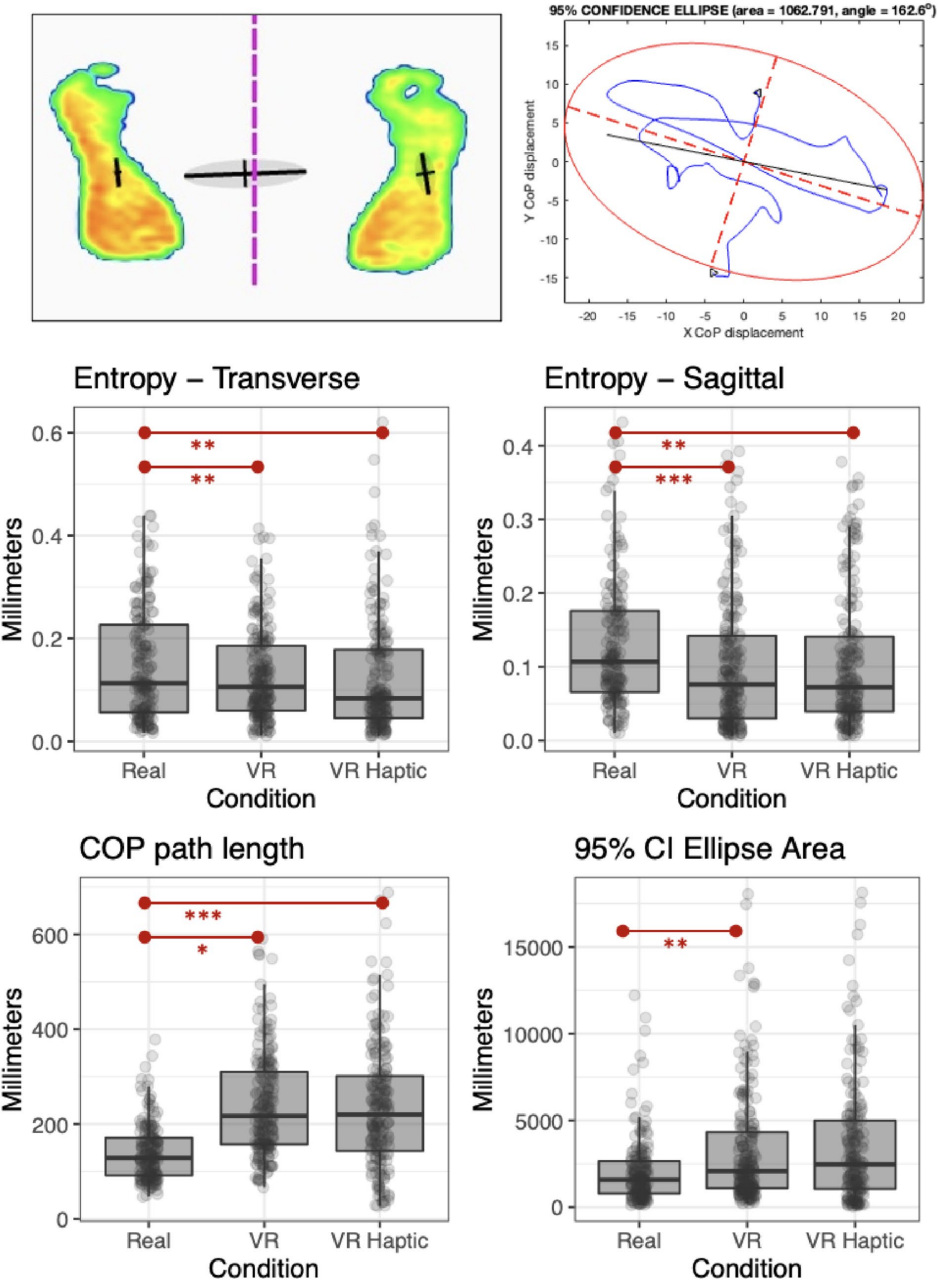
Centre of pressure data. The top left panel shows an example of centre of pressure data being recorded from the pressure plate, with centre of pressure marked for each foot and overall. Top right shows an example of the COP path length for a single trial, and the 95%CI ellipse of the path. Lower panesl show boxplots with overlaid data points for postural control data. *p < .05; **p < .01;***p < .001

A general linear mixed model (Gamma family) with participant as a random factor indicated that the effect of both VR (*p* < 0.001; std. beta = 1.75) and VR Haptic (*p* < 0.001; std. beta = 1.73) were statistically significant. Bonferroni-corrected pairwise comparisons indicated higher entropy in Real compared to VR (*p* = 0.002) and VR Haptic (*p* = 0.003), but no difference between VR and VR Haptic (*p* = 1.00) (see Fig. 5).

#### Sagittal

A general linear mixed model (Gamma family) with participant as a random factor indicated that the effect of both VR (*p* < 0.001; std. beta = 2.77) and VR Haptic were statistically significant (*p* = 0.002; std. beta = 2.04). Bonferroni-corrected pairwise comparisons indicated significantly higher entropy for Real compared to VR (*p* < 0.001) and Real compared to VR Haptic (*p* = 0.004), but no difference between VR and VR Haptic (*p* = 0.55) (see Fig. 5, top).

### COP path length

A linear mixed model with condition and participant as random effects indicated that the effect of both VR (*p* < 0.001; std. beta = 0.95) and VR Haptic (*p* = 0.002; std. beta = 0.76,) was statistically significant and represented large effects. Bonferroni-corrected pairwise comparisons indicated longer COP path lengths for VR (*p* = 0.03) and VR Haptic (*p* < 0.001) compared to real world, but no difference between the two VR conditions (*p* = 0.87) (see Fig. 5).

### COP 95% CI ellipse area

A linear mixed model predicting log transformed area ellipse values with participant as random effect indicated that the effect of VR was statistically significant (*p* < 0.001; std. beta = 0.22) but VR Haptic was not (*p* = 0.08; std. beta = 0.20). Bonferroni corrected contrasts confirmed a larger ellipse area in VR compared to real (*p* = 0.006) but not between real and VR Haptic (*p* = 0.33) or between the two VR conditions (*p* = 0.67).

### Conscious movement processing

**Fig. 6:**
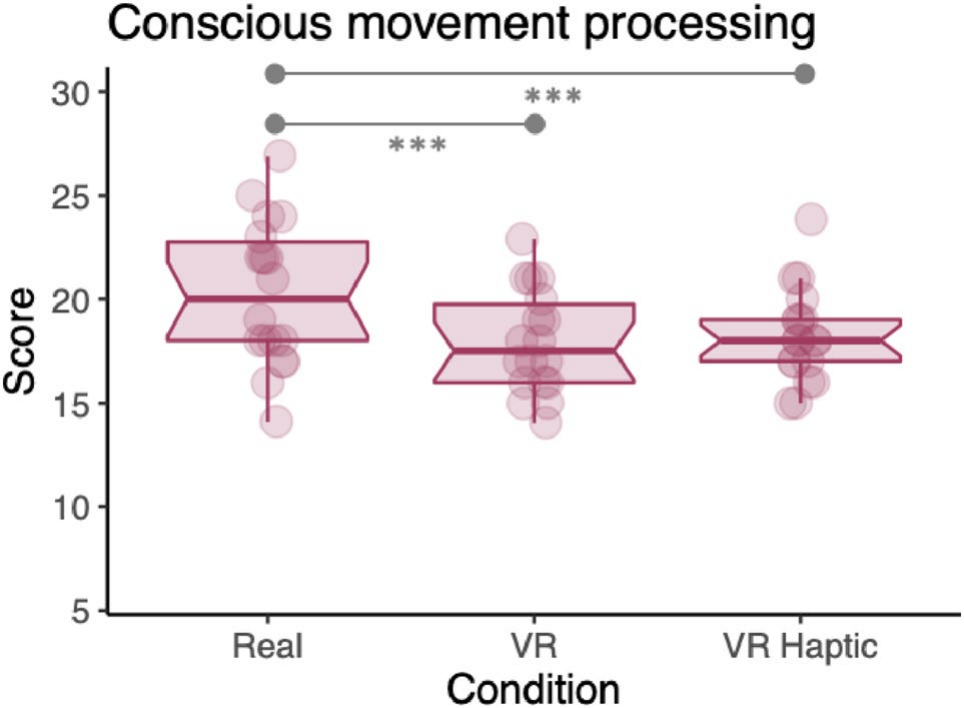
Boxplot with overlaid data points for conscious movement processing questionnaire

We fitted a LMM to predict conscious movement processing (see Fig. 6; Table 1), with participant as a random factor. Within this model, the effect of both VR (*p* < 0.001; std. beta =  – 0.79) and VR Haptic was statistically significant (*p* < 0.001; std. beta = -0.70). Bonferroni-corrected pairwise comparisons indicated significantly higher CMP in real world compared to VR (*p* < 0.001) and in real world compared to VR Haptic (*p* < 0.001). There was no significant difference between the two VR conditions (*p* = 1.00).

## Discussion

Virtual reality poses a novel problem for our sensorimotor system, as it replaces our normal sensory inputs with computer generated ones. It is unclear how much, and in what way, this affects the control of goal-directed movement. Previous research on reach-to-grasp movements has suggested that real world reaches without haptic feedback (Goodale et al. 1994; Whitwell et al. 2015) and reaches in VR (Viau et al. 2004; Gerig et al. 2018; Furmanek et al. 2019) tend to be slower, more exaggerated, and consciously controlled. We aimed to extend this work to a more complex movement, golf putting, to test whether all movements tend to be slower and more consciously controlled. In short, our results suggested that VR putting swings tended to be more variable, more exaggerated, and slower at contact when haptic feedback was absent. Postural control variables indicated more postural movements in VR and that those movements may be more consciously controlled (reduced complexity). Self-reported conscious awareness of the movement was not, however, increased in VR but was decreased. We discuss these findings and their implications in more detail below.

Firstly, we examined the summary measures of putter head control. Higher transverse accelerations at impact were found for real world compared to VR, which partially supported the hypothesis that movements in VR tend to be slower. However, the finding that VR Haptic was also higher than VR (and no different from real world) suggests that this could be due to the presence/absence of the physical ball, rather than due to other features of VR such as the visual presentation. This finding also suggests the addition of haptics in the mixed reality paradigm may have elicited more realistic downswings, and aligns with the findings of Whitwell et al. (2020) that the addition of haptic information can quickly enable participants to switch back to a natural mode of action control. Greater accelerations of the clubhead in the sagittal plane are likely to be detrimental to the execution of the putt as they show that the clubhead was hitting across rather than making a clean contact with the ball (Mackenzie and Evans 2010; Sim and Kim 2010). Greater accelerations of this nature were observed for both VR conditions relative to the real world, suggesting an impairment of swing execution in VR, which was likely due to the visual presentation (as no VR v VR Haptic differences were present). Not only were sagittal accelerations at impact greater in the VR conditions, but the variance over the whole swing was also greater in both VR conditions.

The subsequent SPM analysis indicated different movement profiles for the transverse plane of the swing between real and VR conditions. Figure 3 (top middle panel) suggests that both the backswing and downswing of the club was more exaggerated for both VR conditions relative to the real world. There were no differences in the sagittal plane. As the putter was exactly matched between conditions (and there were no differences between VR and VR Haptic) this suggests that differences in the visual presentation of the environment generated different swing patterns.

Analyses of postural control also revealed important differences between real and VR conditions. Firstly, some small differences in postural sway between real and VR conditions were found, but they did not persist after correction for multiple comparisons. There were, however, clear differences in the total movement of the COP during the putting trials. COP path lengths were longer for both VR conditions relative to the real world, and 95%CI ellipses covered a wider area for VR compared to the real world. This shows greater postural movement in VR. Crucially, higher entropy values also indicated reduced complexity of COP for both VR conditions. This suggests simpler and more regular postural adjustments in VR, characteristic of less automatic (i.e., more consciously processed) postural control (Borg and Laxåback 2010), in line with our predictions. The absence of differences between the two VR conditions indicates that the control of posture during the putt was not affected by the presence/absence of haptic feedback and instead other features of VR were responsible for the postural changes. Further investigations into the exact cause of these differences in balance control may require more closely matched environmental features, in case background visual information (e.g., scenery) could influence balance.

Our findings point to haptic information as the source of some of the movement differences in VR but suggest that other factors (e.g., visual features) might be responsible for other disparities. For instance, for several of the putting kinematic variables (e.g., transverse accelerations and jerk), the VR Haptic condition was more similar to the real world, suggesting that increasingly realistic haptics could remove, or at least minimise, differences between these aspects of real world and VR action control. For the postural control measures, however, there was very little difference between the VR conditions, indicating that haptic information was not the cause and visual features of the VR environment such as distortions of space or vergence-accommodation conflict were most likely responsible. We cannot, however, draw definitive conclusions about the origins of these differences. Indeed, some differences could even be independent of perceptual distortions and instead simply due to the participant’s knowledge that the environment they are in is not real. Consequently, it will be crucial for future work to further unpick the source of these observed differences.

In stark contrast to our prediction, self-reported conscious processing of movement was not higher in the two VR conditions. In fact, CMP scores were observed to be higher in the real-world condition than either VR or VR Haptic (see Fig. 6). This result seems to conflict with the observed decrease in postural sway complexity signalling more consciously controlled balance. While unexpected, this effect could plausibly be an artefact of the absence of any body visualization in our VR tasks. Previous work has examined how the presence or absence of a visual representation of the body in VR can affect the sense of ‘embodiment’ in VR (Kilteni et al. 2012), which refers to the user’s perception of the avatar as collocated with their physical body, and that they own the body. The lack of an avatar might have meant a lack of embodiment and reduced awareness of the body and subsequently reduced the perception of ‘inclusion’ in the virtual environment (Sallnäs et al. 2000). Body visualization has been found to impact performance in sport-related VR tasks (Pastel et al. 2020) so comparisons of tasks with and without body visualizations could be important in future work. Speculatively, this reduction in bodily awareness could present an opportunity for promoting more implicit motor skill learning in VR and reducing subsequent risk of reinvestment (e.g., see (Maxwell et al. 2000)). This effect may be particularly beneficial to persons with a high propensity for conscious movement processing (Zhu et al. 2011).

## Conclusion

In summary, VR and related immersive technologies offer new opportunities for learning motor skills in more flexible ways, but there is still a significant gap in our understanding of whether skills performed in VR are similar to those in the real world (Harris et al. 2019). As such, applications to sport and rehabilitation could be disrupted without a better understanding. Here, we found that there were clear differences in the execution of a visuomotor skill when performed in the real world, in VR, and in VR Haptic conditions. While the addition of haptic information in VR brought swing kinematics more in line with the real world, there were persistent postural control differences in VR. There results supported the broad hypothesis that movements in VR tend to be more exaggerated and consciously controlled. Further work is, however, needed to determine which particular features of VR (e.g., impoverished haptics, missing depth cues, reduced field of view) are directly responsible for these differences.

## Supplementary Information

Below is the link to the electronic supplementary material.

Supplementary file1 (DOCX 15 KB)

## Data Availability

All relevant data and code are available online from: https://osf.io/h8az7/.

## Footnotes

1 Model summaries are reported in Table 1, only effect sizes and significance values for the main effects are reported in the text.
