# Supplementary material for: Movement kinematic and postural control differences when performing a visuomotor skill in real and virtual environments
Source: Exp Brain Res. 2023 May 24;241(7):1797–810. doi: 10.1007/s00221-023-06639-0 (PMC10348942; doi:10.1007/s00221-023-06639-0)
Supplement: Supplementary file 1 — Supplementary file1 (DOCX 15 KB) [file 221_2023_6639_MOESM1_ESM.docx]

For simplicity we have not re-reported the effect of VR/VR Haptic in each model. We have highlighted in red any significant effects.

**Downswing acceleration at impact**

***Transverse***.

We fitted a linear mixed model to predict transvers accelerations at impact with Condition and Trial. The model's total explanatory power is moderate (conditional R^2^ = 0.23) and the part related to the fixed effects alone (marginal R^2^) is of 0.06. Within this model:

- The effect of Trial is statistically non-significant and negative (beta = -7.38e-03, 95% CI [-0.06, 0.04], t(928) = -0.28, p = 0.782; Std. beta = -0.02, 95% CI [-0.14, 0.10])

- The interaction effect of Trial on Condition [VR] is statistically non-significant and negative (beta = -0.02, 95% CI [-0.09, 0.04], t(928) = -0.67, p = 0.502; Std. beta = -0.05, 95% CI [-0.20, 0.10])

- The interaction effect of Trial on Condition [VR Haptic] is statistically non-significant and negative (beta = -0.06, 95% CI [-0.13, 8.80e-03], t(928) = -1.71, p = 0.088; Std. beta = -0.14, 95% CI [-0.29, 0.02])

***Sagittal***.

We fitted a linear mixed model to predict sagittal accelerations at impact with Condition and Trial. The model's total explanatory power is substantial (conditional R^2^ = 0.56) and the part related to the fixed effects alone (marginal R^2^) is of 0.05. Within this model:

- The effect of Trial is statistically non-significant and negative (beta = -9.83e-03, 95% CI [-0.03, 0.01], t(923) = -0.87, p = 0.383; Std. beta = -0.04, 95% CI [-0.14, 0.06])

- The interaction effect of Trial on Condition [VR] is statistically non-significant and positive (beta = 0.02, 95% CI [-9.50e-03, 0.04], t(923) = 1.28, p = 0.202; Std. beta = 0.08, 95% CI [-0.04, 0.20])

- The interaction effect of Trial on Condition [VR Haptic] is statistically significant but small (beta = 0.03, 95% CI [5.10e-03, 0.06], t(923) = 2.32, p = 0.021; Std. beta = 0.15, 95% CI [0.02, 0.27]).

**Sagittal variance**

We fitted a linear mixed model to predict sagittal variance with Condition and Trial. The model's total explanatory power is substantial (conditional R^2^ = 0.51) and the part related to the fixed effects alone (marginal R^2^) is of 0.11. Within this model:

- The effect of Trial is statistically significant but very small (beta = -0.02, 95% CI [-0.04, -3.15e-03], t(919) = -2.29, p = 0.022; Std. beta = -0.04, 95% CI [-0.08, 8.01e-03])

- The interaction effect of Trial on Condition [VR] is statistically significant but very small (beta = 0.04, 95% CI [0.01, 0.06], t(919) = 3.16, p = 0.002; Std. beta = 0.07, 95% CI [0.02, 0.13])

- The interaction effect of Trial on Condition [VR Haptic] is statistically significant but very small (beta = 0.04, 95% CI [0.01, 0.06], t(919) = 3.12, p = 0.002; Std. beta = 0.09, 95% CI [0.03, 0.14])

**Jerk**

***Transverse*.**

We fitted a linear mixed model to predict transverse jerk with Condition and Trial. The model's total explanatory power is substantial (conditional R^2^ = 0.48) and the part related to the fixed effects alone (marginal R^2^) is of 0.08. Within this model:

- The effect of Trial is statistically significant and negative (beta = -0.14, 95% CI [-0.24, -0.04], t(922) = -2.74, p = 0.006; Std. beta = -0.16, 95% CI [-0.27, -0.04])

- The interaction effect of Trial on Condition [VR] is statistically significant and positive (beta = 0.15, 95% CI [0.02, 0.27], t(922) = 2.31, p = 0.021; Std. beta = 0.16, 95% CI [0.02, 0.30])

- The interaction effect of Trial on Condition [VR Haptic] is statistically non-significant and positive (beta = 0.08, 95% CI [-0.04, 0.21], t(922) = 1.30, p = 0.194; Std. beta = 0.09, 95% CI [-0.05, 0.23])

***Sagittal*.**

We fitted a linear mixed model to predict sagittal jerk with Condition and Trial. The model's total explanatory power is substantial (conditional R^2^ = 0.36) and the part related to the fixed effects alone (marginal R^2^) is of 0.02. Within this model:

- The effect of Trial is statistically non-significant and negative (beta = -0.02, 95% CI [-0.06, 0.02], t(920) = -0.98, p = 0.328; Std. beta = -0.06, 95% CI [-0.19, 0.06])

- The interaction effect of Trial on Condition [VR] is statistically non-significant and positive (beta = 0.02, 95% CI [-0.02, 0.07], t(920) = 0.95, p = 0.341; Std. beta = 0.07, 95% CI [-0.08, 0.23])

- The interaction effect of Trial on Condition [VR Haptic] is statistically non-significant and positive (beta = 0.03, 95% CI [-0.01, 0.08], t(920) = 1.45, p = 0.148; Std. beta = 0.11, 95% CI [-0.04, 0.27])

**Postural Sway Amplitude**

***Transverse***.

We fitted a linear mixed model to predict Transverse sway amplitude with Condition and Trial. The model's total explanatory power is substantial (conditional R^2^ = 0.51) and the part related to the fixed effects alone (marginal R^2^) is of 0.05. Within this model:

- The effect of Trial is statistically non-significant and positive (beta = 0.20, 95% CI [-1.80e-03, 0.40], t(567) = 1.95, p = 0.052; Std. beta = 0.10, 95% CI [-9.05e-04, 0.20])

- The interaction effect of Trial on Condition [VR] is statistically non-significant and negative (beta = -0.02, 95% CI [-0.30, 0.27], t(567) = -0.11, p = 0.911; Std. beta = -8.21e-03, 95% CI [-0.15, 0.14])

- The interaction effect of Trial on Condition [VR Haptic] is statistically non-significant and positive (beta = 0.18, 95% CI [-0.13, 0.49], t(567) = 1.13, p = 0.260; Std. beta = 0.09, 95% CI [-0.07, 0.25])

***Sagittal.***

We fitted a linear mixed model to predict sagittal sway amplitude with Condition and Trial. The model's total explanatory power is substantial (conditional R^2^ = 0.47) and the part related to the fixed effects alone (marginal R^2^) is of 0.04. Within this model:

- The effect of Trial is statistically non-significant and positive (beta = 0.09, 95% CI [-0.05, 0.23], t(567) = 1.30, p = 0.195; Std. beta = 0.07, 95% CI [-0.04, 0.18])

- The interaction effect of Trial on Condition [VR] is statistically non-significant and positive (beta = 5.16e-03, 95% CI [-0.19, 0.20], t(567) = 0.05, p = 0.959; Std. beta = 3.98e-03, 95% CI [-0.15, 0.16])

- The interaction effect of Trial on Condition [VR Haptic] is statistically non-significant and negative (beta = -8.80e-03, 95% CI [-0.22, 0.21], t(567) = -0.08, p = 0.936; Std. beta = -6.79e-03, 95% CI [-0.17, 0.16])

**Postural Sway Complexity**

***Transverse***.

We fitted a linear mixed model to predict SampEn in the transverse plance with Condition and Trial. The model's total explanatory power is substantial (conditional R^2^ = 0.36) and the part related to the fixed effects alone (marginal R^2^) is of 0.03. Within this model:

- The effect of Trial is statistically non-significant and negative (beta = -4.66e-04, 95% CI [-2.57e-03, 1.64e-03], t(567) = -0.43, p = 0.664; Std. beta = -0.03, 95% CI [-0.14, 0.09])

- The interaction effect of Trial on Condition [VR] is statistically non-significant and negative (beta = -2.11e-03, 95% CI [-5.14e-03, 9.14e-04], t(567) = -1.37, p = 0.171; Std. beta = -0.11, 95% CI [-0.28, 0.05])

- The interaction effect of Trial on Condition [VR Haptic] is statistically non-significant and negative (beta = -1.21e-03, 95% CI [-4.47e-03, 2.05e-03], t(567) = -0.73, p = 0.466; Std. beta = -0.07, 95% CI [-0.24, 0.11])

***Sagittal.***

We fitted a linear mixed model to predict SampEn in the sagittal plance with Condition and Trial. The model's total explanatory power is substantial (conditional R^2^ = 0.38) and the part related to the fixed effects alone (marginal R^2^) is of 0.04. Within this model:

- The effect of Trial is statistically significant but small (beta = -2.71e-03, 95% CI [-5.09e-03, -3.23e-04], t(567) = -2.23, p = 0.026; Std. beta = -0.13, 95% CI [-0.24, -0.02])

- The interaction effect of Trial on Condition [VR] is statistically non-significant and positive (beta = 1.67e-03, 95% CI [-1.76e-03, 5.09e-03], t(567) = 0.96, p = 0.339; Std. beta = 0.08, 95% CI [-0.08, 0.24])

- The interaction effect of Trial on Condition [VR Haptic] is statistically non-significant and positive (beta = 5.34e-04, 95% CI [-3.18e-03, 4.25e-03], t(567) = 0.28, p = 0.777; Std. beta = 0.03, 95% CI [-0.15, 0.20])

**COP Path Length**

We fitted a linear mixed model to predict COP path length with Condition and Trial. The model's total explanatory power is substantial (conditional R^2^ = 0.61) and the part related to the fixed effects alone (marginal R^2^) is of 0.16. Within this model:

- The effect of Trial is statistically non-significant and positive (beta = 0.60, 95% CI [-1.47, 2.66], t(567) = 0.57, p = 0.571; Std. beta = 0.03, 95% CI [-0.06, 0.12])

- The interaction effect of Trial on Condition [VR] is statistically non-significant and positive (beta = 0.71, 95% CI [-2.29, 3.70], t(567) = 0.46, p = 0.643; Std. beta = 0.03, 95% CI [-0.10, 0.16])

- The interaction effect of Trial on Condition [VR Haptic] is statistically non-significant and positive (beta = 1.04, 95% CI [-2.21, 4.28], t(567) = 0.63, p = 0.531; Std. beta = 0.04, 95% CI [-0.10, 0.19])

**COP 95% CI Ellipse Area**

We fitted a linear mixed model to predict COP ellipse area with Condition and Trial. The model's explanatory power related to the fixed effects alone (marginal R^2^) is 0.10. Within this model:

- The effect of Trial is statistically non-significant and positive (beta = 42.54, 95% CI [-21.37, 106.45], t(567) = 1.31, p = 0.192; Std. beta = 0.07, 95% CI [-0.03, 0.17])

- The interaction effect of Trial on Condition [VR] is statistically non-significant and negative (beta = -15.50, 95% CI [-108.79, 77.78], t(567) = -0.33, p = 0.744; Std. beta = -0.02, 95% CI [-0.17, 0.12])

- The interaction effect of Trial on Condition [VR Haptic] is statistically non-significant and positive (beta = 81.53, 95% CI [-19.62, 182.69], t(567) = 1.58, p = 0.114; Std. beta = 0.13, 95% CI [-0.03, 0.29])
